# Altered Expression Ratio of Actin-Binding Gelsolin Isoforms Is a Novel Hallmark of Mitochondrial OXPHOS Dysfunction

**DOI:** 10.3390/cells9091922

**Published:** 2020-08-19

**Authors:** Alberto García-Bartolomé, Ana Peñas, María Illescas, Verónica Bermejo, Sandra López-Calcerrada, Rafael Pérez-Pérez, Lorena Marín-Buera, Cristina Domínguez-González, Joaquín Arenas, Miguel A. Martín, Cristina Ugalde

**Affiliations:** 1 Laboratorio de Enfermedades Mitocondriales y Neurometabólicas, Instituto de Investigación Hospital 12 de Octubre (i+12), 28041 Madrid, Spain; alberto7188@hotmail.com (A.G.-B.); anappdv.imas12@h12o.es (A.P.); maria.illescas@hotmail.com (M.I.); vbermejo.imas12@h12o.es (V.B.); sandra.lopezcal@gmail.com (S.L.-C.); rafaperez44@gmail.com (R.P.-P.); Lorena.marin@biolamina.com (L.M.-B.); c.dom.glez@gmail.com (C.D.-G.); joaquin.arenas@salud.madrid.org (J.A.); mamcasanueva.imas12@h12o.es (M.A.M.); 2 Centro de Investigación Biomédica en Red de Enfermedades Raras (CIBERER), U723 Madrid, Spain; 3 Neuromuscular Unit, Hospital Universitario 12 de Octubre, 28041 Madrid, Spain

**Keywords:** mitochondria, OXPHOS dysfunction, oxidative stress, actin cytoskeleton, gelsolin isoforms, GSN, biomarker

## Abstract

Mitochondrial oxidative phosphorylation (OXPHOS) defects are the primary cause of inborn errors of energy metabolism. Despite considerable progress on their genetic basis, their global pathophysiological consequences remain undefined. Previous studies reported that OXPHOS dysfunction associated with complex III deficiency exacerbated the expression and mitochondrial location of cytoskeletal gelsolin (GSN) to promote cell survival responses. In humans, besides the cytosolic isoform, GSN presents a plasma isoform secreted to extracellular environments. We analyzed the interplay between both GSN isoforms in human cellular and clinical models of OXPHOS dysfunction. Regardless of its pathogenic origin, OXPHOS dysfunction induced the physiological upregulation of cytosolic GSN in the mitochondria (mGSN), in parallel with a significant downregulation of plasma GSN (pGSN) levels. Consequently, significantly high mGSN-to-pGSN ratios were associated with OXPHOS deficiency both in human cells and blood. In contrast, control cells subjected to hydrogen peroxide or staurosporine treatments showed no correlation between oxidative stress or cell death induction and the altered levels and subcellular location of GSN isoforms, suggesting their specificity for OXPHOS dysfunction. In conclusion, a high mitochondrial-to-plasma GSN ratio represents a useful cellular indicator of OXPHOS defects, with potential use for future research of a wide range of clinical conditions with mitochondrial involvement.

## 1. Introduction

Mitochondria participate in a large variety of metabolic and physiological processes, such as lipid metabolism, iron-sulphur cluster biogenesis, hormone and reactive oxygen species (ROS) signaling, calcium buffering, and apoptosis, and importantly, they provide most of the energy (ATP) usable by cells through the oxidative phosphorylation (OXPHOS) system. The OXPHOS system is located in the mitochondrial inner membrane, and it is composed of five multiprotein enzyme complexes and two mobile electron carriers (ubiquinone and cytochrome *c*) that couple respiration to ATP synthesis: Complexes I to IV (CI-CIV) constitute the mitochondrial respiratory chain (MRC), which transfers electrons from NADH and FADH_2_ to molecular oxygen, with a parallel generation of a proton gradient across the mitochondrial inner membrane that is used by complex V for ATP synthesis [1]. Primary functional defects of the OXPHOS machinery are the leading cause of inborn errors of energy metabolism, involving more than 350 disorder-related genes [2]. Notably, OXPHOS dysfunction is likely involved in the pathogenesis of common clinical conditions, such as obesity and diabetes [3,4,5]; neurodegenerative conditions, like Alzheimer’s or Parkinson’s disease [6,7]; and may be crucial in cancer treatment [8,9]. Despite the wide knowledge on the molecular genetic origins of OXPHOS disorders [10], their underlying pathophysiological mechanisms remain mostly unknown. The diagnosis of OXPHOS diseases remains challenging due to their wide clinical heterogeneity, and research for specific and sensitive diagnostic tools based on serum biomarkers is currently active [10].

High-resolution differential proteomics applied to the diagnosis of OXPHOS deficiency previously identified potential therapeutic targets for mitochondrial disorders [11,12]. Particularly, analyses in primary skin fibroblasts from patients with mutations in *BCS1L*, encoding an assembly factor involved in the biogenesis of MRC complex III [13,14], revealed a protein profile showing main alterations in energy metabolism, as well as in the structural organization of the cytoskeleton, regulation of gene expression, protein transport, and metabolic stress responses [12]. The most significantly upregulated protein was the cytosolic isoform of gelsolin (GSN), an ~81-kDa cytoskeletal protein that regulates the cap and severing of actin filaments in a calcium-dependent fashion [15]. Besides its role in actin filaments’ remodeling, GSN plays a multifunctional regulatory role as a transcriptional coactivator, as well as a downstream effector of cell signaling on the crosstalk between the actin cytoskeleton and transmembrane receptors [16,17]. In humans, besides the cytosolic GSN isoform, there is a plasma isoform (pGSN) mainly secreted from muscle into the extracellular space, which can be easily detected in serum or plasma [18,19,20]. The human pGSN isoform, ~86 kDa, contains a specific secretory signal peptide of 51 amino acids at its N-terminus (UniProtKB references P06396 and P06396-2), and a disulphide bond that confers additional stability in the extracellular environment [21]. pGSN plays a relevant role in cardiovascular disease, as it is part of the extracellular actin scavenger system (EASS) responsible for rapid severing and removal of actin filaments released from tissue necrosis into the bloodstream, by sequestering monomeric actin and increasing its clearance from the circulation [22,23]. Both isoforms are encoded by alternative splicing of *GSN* mRNAs, and maintain their ability to depolymerize actin filaments [24].

Mutations in the *GSN* gene have been linked to familial amyloidosis of Finnish type (FAF) or gelsolin amyloidosis [MIM#105120], a rare, autosomal dominant hereditary amyloid polyneuropathy, mainly characterized by progressive cranial neuropathies, corneal lattice dystrophy, and sensory neuropathy [25]. This disorder is caused by the systemic aggregation and deposition of aberrant proteolytic fragments of pGSN mediated by matrix metalloproteases [26]. Moreover, altered expression of both GSN isoforms has been associated to a diversity of pathophysiological conditions. Due to its role in EASS, decreased pGSN levels result in detrimental effects caused by actin accumulation in the bloodstream: Increased blood viscosity affecting microvascular flow, platelet activation and aggregation, microvascular thrombosis, release of proinflammatory mediators, fibrinolysis impairment, and increased alpha-haemolysin production [24]. Thus, decreased pGSN levels were consistently reported in cardiovascular diseases, major trauma and injuries, diabetes [27], and in other relevant pathologies, such as Alzheimer’s disease, rheumatoid arthritis, sepsis, liver failure, or cancer [17,24,28], to the point that pGSN has been proposed as a general biomarker of health prognosis [28]. In contrast, upregulation of cytosolic GSN expression mainly influences cytoskeletal turnover and dynamics, and was associated with ageing [29], Down syndrome [30], or heart failure [31,32]. Experimental evidence suggests that increased cytoplasmic GSN levels are triggered under oxidative stress conditions, such as lipid peroxidation [33,34], in the presence of calcium ionophores [35], upon hydrogen peroxide treatment via PKC activators [36], as well as in mouse HIF-1 knock-out (KO) fibroblasts subjected to hypoxia [37]. Studies in cancer cells showed that cytosolic GSN expression may impact the cellular redox milieu and cell survival by modulating intracellular O^2.−^/H_2_O_2_ levels, possibly by the interaction and suppression of superoxide dismutase 1 (Cu/Zn SOD) enzymatic activity [38,39]. Other studies in human cell lines with MRC complex III deficiency demonstrated the upregulation and location of GSN at the mitochondrial outer membrane (henceforth mitochondrial GSN or mGSN), where it interacts with the voltage-dependent anion channel (VDAC) to prevent the release of mitochondrial cytochrome *c* into the cytosol and apoptotic cell death [40]. However, whether these GSN-mediated survival adaptations occur as a general response to oxidative stress or are specific to OXPHOS dysfunction remains largely unknown.

In this work, we analyzed the interplay between the cytosolic and plasma isoforms of GSN in human cellular models of OXPHOS deficiency and oxidative stress. Our results show that a high mitochondrial-to-plasma GSN ratio represents a useful indicator of OXPHOS system dysfunction in human cultured cells. Importantly, the mGSN:pGSN ratio was modulated by genetic manipulation of OXPHOS-deficient cell lines. These analyses were extrapolated to blood from patients with OXPHOS disorders, representing a promising proof of concept for future research on the diagnosis of these devastating pathologies.

## 2. Materials and Methods

### 2.1. Cell Cultures and Treatments

Primary skin fibroblasts were obtained from healthy donors (age and sex matched) and pediatric patients (P1–P4) with mutations in *BCS1L* that displayed MRC complex III deficiency [11]. Fibroblasts were cultured in 1 g/L glucose-containing Dulbecco′s modified Eagle′s Medium (DMEM) (Invitrogen, ThermoFisher, UT, USA) supplemented with 10% fetal bovine serum (FBS), 100 IU/mL penicillin, and 100 IU/mL streptomycin at 37 °C and 5% CO_2_. Written informed consent was previously obtained from the parents of affected children.

Detailed pathophysiological characterization of OXPHOS deficiency was previously performed on the transmitochondrial cybrids and HEK293T cells used in our study [41,42,43,44,45,46,47]. The complex I-deficient cybrids (CI-KD) harbor a homoplasmic m.4681T>C mutation in the *MT-ND2* subunit gene that leads to a severe complex I assembly defect due to a p.L71P substitution [41]. The complex III mutant cell line (CIII-KO) contains a homoplasmic 4-base pair deletion in the *MT-CYB* gene that affects the de novo synthesis of cytochrome *b* and completely abolishes complex III activity [42,43]. The first complex IV mutant cell line (CIV-KO1) lacks CIV due to the homoplasmic m.6930G>A transition in the *MT-CO1* gene, which generates a stop codon leading to the loss of the last 170 amino acids of the COX1 polypeptide [44]. The second complex IV mutant cell line (CIV-KO2) lacks functional CIV due to the homoplasmic m.7896 G>A transition in the *MT-CO2* gene, creating a stop codon that results in the loss of 123 amino acids at the C-terminus of the COX2 polypeptide [45]. The complex IV-deficient COX18-KO HEK293T cells were previously generated by TALEN-based gene editing [47]. Transmitochondrial cybrids, control 143B-TK^−^ cells, and HEK293T cells were cultured in 4.5 g/L glucose-containing DMEM medium (Invitrogen) supplemented with 10% FBS, 50 μg/mL uridine, and 100 IU/mL penicillin/streptomycin.

To induce a general blockage of OXPHOS function, cells were treated for up to 48 h with the MRC complex III activity inhibitor antimycin A (AA) at concentrations ranging between 2 and 500 nM. To analyze the effect of oxidative stress on GSN levels, control 143B-TK^−^ cells were treated with 100, 200, and 500 µM hydrogen peroxide (H_2_O_2_, Merck, Darmstadt, Germany) for 6, 24, and 36 h before harvesting, as previously described [36]. To induce apoptotic cell death, control cells were treated with 1 and 2 μM staurosporine for 6, 15, and 24 h. In all experiments, cells were balanced for passage number.

### 2.2. Blood Samples

Blood samples were collected in EDTA anticoagulant tubes from 9 adult patients (P5–P13, Table 1) who were clinically, biochemically, and/or genetically diagnosed with MRC deficiency at Hospital Universitario 12 de Octubre (Madrid, Spain), as well as from 9 healthy individuals (C1–C9, Table 2). This study was approved by the institutional Ethics Committee at Hospital Universitario 12 de Octubre, and it is in accordance with the Declaration of Helsinki for Human Research.

### 2.3. Peripheral Blood Mononuclear Cells and Plasma Separation

Peripheral blood mononuclear cells (PBMCs) were extracted using Ficoll-Paque PLUS density centrifugation (GE Healthcare, Barcelona, Spain). In total, 3 mL of Ficoll-Paque media were added to a centrifuge tube and 6 mL of diluted blood samples were carefully layered onto the Ficoll-Paque media solution without mixing. Next, the tube was centrifuged at 750 *g* for 20 min at RT (brake turned off). The upper layer containing plasma and platelets was drawn off and centrifuged at 2000 *g* for 10 min. Supernatant (plasma) was aliquoted and immediately stored at −80 °C. The undisturbed layer corresponding to mononuclear cells was transferred to a sterile centrifuge tube; PBMCs were washed with phosphate-buffered saline (PBS), aliquoted, and stored at −80 °C.

### 2.4. Mitochondrial Respiration

Basal oxygen consumption rates were measured with an XF24 Extracellular Flux Analyzer (Seahorse Bioscience, Agilent, CA, USA). The day before the experiment, around 9 × 10^4^ cells per well were seeded in order to obtain 150–200 µg of protein per well on the day of the experiment. Cells were then incubated for 1 h in unbuffered DMEM supplemented with 4.5 g/L glucose (cybrids) or 1 g/L glucose (fibroblasts), 1 mM sodium pyruvate, and 2 mM glutamine at 37 °C in a CO_2_-free incubator. Initially, baseline cellular OCR was measured, and mitochondrial basal respiration was calculated by subtracting non-mitochondrial respiration upon addition of the mitochondrial inhibitors rotenone and antimycin A at a 1 μM final concentration. In all experiments, the protein concentration per well was determined using the Pierce BCA Protein Assay Kit (ThermoFisher, IL, USA) after cell lysis in extraction buffer, and was used to calibrate the oxygen consumption data (pmol/min/mg).

### 2.5. Intracellular ROS Levels

Concentrations of reactive oxygen species (ROS) were determined with the Amplex Red Hydrogen Peroxide/Peroxidase Assay kit (Molecular Probes, ThermoFisher, UK), according to the manufacturer′s guidelines. Briefly, cybrid cultures were incubated for 30 min at 37 °C in the dark with a reaction mix containing the Amplex red reagent and HRP peroxidase. Absorbance was measured at 560 nm in an Epoch Microplate Spectrophotometer (Biotek, VT, USA), and values were normalized for the total amount of protein per well.

### 2.6. Isolation of Mitochondrial Fractions from Human Cells

Cultured cells and PBMCs were harvested and mitochondrial fractions were isolated with the Mitochondria Isolation Kit human MACS (Miltenyi Biotec, Gladbach, Germany), according to the manufacturer′s guidelines.

### 2.7. Purification of Secreted Proteins from Cultured Cells

Cells were cultured for 24 h in DMEM medium without FBS supplementation. Extracellular media were harvested and the secreted protein extracts (secretomes) were concentrated using Amicon Ultra-15 tubes (Merck, Darmstadt, Germany) according to the manufacturer′s guidelines. Briefly, secretomes were centrifuged at 4000 *g* for 10 min, and the collected fraction was washed three times with cold PBS and resuspended in 100 µL of extraction buffer (20 mM HEPES NaOH pH 7.4, 150 mM NaCl, 10% glycerol, 1% Tx-100) and Protease Inhibitor Cocktail Tablets (Roche, Basel, Switzerland).

### 2.8. SDS-PAGE Electrophoresis

Protein concentrations were measured using the micro BCA protein assay (ThermoFisher, IL, USA). Whole-cell and mitochondrial protein extracts (15 µg) were resolved on conventional 10% SDS-PAGE gels and transferred to PROTRAN^®®^ nitrocellulose membranes (Whatman GmbH, GE Healthcare Life Sciences, Germany). Secreted protein extracts (25 µg) were separated either on conventional 10% SDS-PAGE gels and visualized by Coomassie staining (Fisher Bioreagents, Geel, Belgium), or, alternatively, they were resolved on 10% Mini-Protein TGX Stain Free Precast Gels (Bio-Rad, Mory, France) followed by quantification of the fluorescent protein signals in a ChemiDoc™ MP Imager (Bio-Rad, CA, USA).

### 2.9. Blue Native Electrophoresis and In-Gel Activity Assays

Mitochondrial pellets were isolated from T_175_ cell culture flasks or 15-cm Petri dishes (ThermoFisher, Madrid, Spain). Pre-cast NativePAGE^TM^ 3–12% Bis-Tris gels (ThermoFisher, UK) were loaded with 60–80 μg of mitochondrial protein and processed for blue native electrophoresis (BN-PAGE) and in-gel activity (IGA) assays as described before [48]. After electrophoresis, proteins were transferred to nitrocellulose membranes at 40 V overnight and probed with antibodies.

### 2.10. Antibody Detection

Immunoblotting was performed with primary antibodies raised against human GSN, complex I NDUFA9 subunit, complex II SDHA subunit, complex III CORE2 subunit (Abcam, Cambridge, UK), complex IV COX5B subunit (Santa Clara, TX, USA), or μ-Actin (Sigma). Peroxidase-conjugated anti-mouse or anti-rabbit IgGs (Molecular Probes) were used as secondary antibodies. Immunoreactive bands were detected with ECL Prime Western Blotting Detection Reagent (Amersham Biosciences, UK) in a ChemiDoc™ MP Imager (Bio-Rad), and their optical densities were measured using the ImageLab™ (Bio-Rad) and the ImageJ analysis software.

### 2.11. GSN ELISA Analyses

Plasma GSN (pGSN) concentrations were measured with a specific ELISA kit to GSN (Aviscera Bioscience Inc., Quimigen, CA, USA) in plasma samples previously diluted 1:8000 to 12,000 for pGSN detection, according to the manufacturer′s guidelines.

### 2.12. Data Analysis

All experiments were performed at least in triplicate, unless otherwise indicated, and results were presented as mean ± standard deviation (SD) values. Statistical *p* values were obtained by application of the Mann–Whitney *U* test using GraphPad and SPSS v21.0 programs.

## 3. Results

### 3.1. Cytoplasmic GSN Is Upregulated and Targeted to Mitochondria as a General Response to OXPHOS Deficiency

**Figure 1:**
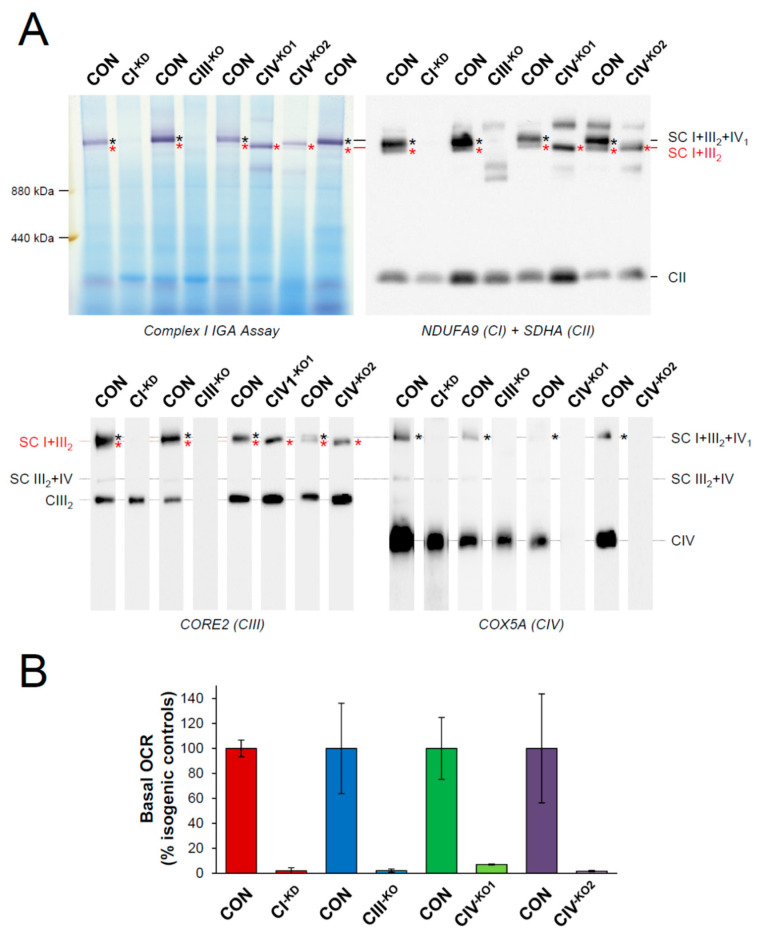
Structural and functional defects of the respiratory chain in mutant transmitochondrial cybrids. (**A**) Mitochondria were isolated from isogenic controls (CON) and mutant cybrids harboring homoplasmic mutations in the mitochondrial *MT-ND2* (CI^−KD^), *MT-CYB* (CIII^−KO^), *MT-CO1* (CIV1^−KO^), and *MT-CO2* (CIV2^−KO^) genes. Digitonized mitochondria (digitonin-to-protein ratio of 4:1) were analyzed by BN-PAGE, and supercomplexes were visualized by complex I in-gel activity (IGA) assay and Western blot with the indicated antibodies that recognize subunits from MRC complexes I-IV. The relative position of the molecular weight marker is indicated on the left of the upper panels. The identity of the respiratory chain supercomplexes (SCs) is I + III_2_ + IV_1_, SC containing complexes I, III_2_ and IV; I + III_2_, SC containing complexes I and III_2_; III_2_ + IV, SC containing complexes III_2_ and IV; III_2_, CIII dimer. (**B**) Mitochondrial basal oxygen consumption rates (OCR) were measured in control (CON) and mutant cybrids respiring in 4.5 g/L glucose-containing medium. Three technical measurements per sample were performed in two independent experiments in the isogenic controls (CON) and the CI^−KD^, CIII^−KO^, and CIV2^−KO^ cell lines, and once in the CIV1^−KO^ cybrids. Data are expressed as the mean ± SD of the percentages of the isogenic control values. OCR values were calculated in pmol O_2_.min^−1^.μg protein^−1^ in all experiments.

Previous studies in mitochondrial complex III (CIII)-deficient cell lines revealed a specific upregulation of cytosolic GSN and its localization to the mitochondrial outer membrane (mGSN), where it interacts with VDAC1 to promote protective antiapoptotic responses [12,40]. To discriminate whether this phenomenon is specific to CIII deficiency, rather than occurring as a general response to OXPHOS enzyme defects, we analyzed control and mutant cybrids with different types of OXPHOS deficiency (Figure 1). All cybrid clones contained the nuclear background from 143B TK^−^ *rho zero* cells [49], and were populated with mitochondria harboring homoplasmic mutations in the CI *MT-ND2*, CIII *MT-CYB*, CIV *MT-CO1*, and CIV *MT-CO2* genes (CI-KD, CIII-KO, CIV-KO1, and CIV-KO2, respectively) [41,44,45,50]. Blue native electrophoresis followed by CI-IGA assay or by Western blot with different antibodies recognizing specific subunits from complexes I-IV (Figure 1A) revealed severe structural alterations of the MRC complexes I, III, or IV and of the mitochondrial supercomplexes (SCs) in the mutant cybrids relative to their isogenic controls. Mitochondrial basal respiration analyses (Figure 1B) confirmed the previously described functional abolishment of the OXPHOS system as a consequence of the structural loss of functional MRC complexes I, III, or IV in the mutants [41,42,43,51,52].

**Figure 2:**
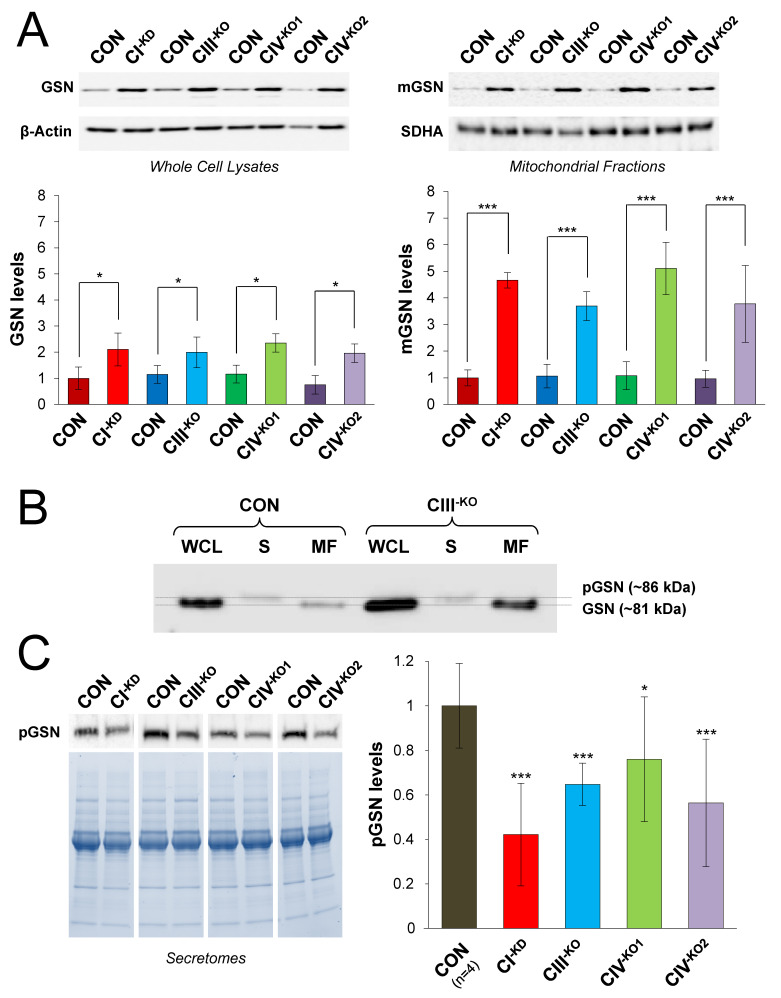
Relative expression of gelsolin isoforms in mitochondrial and secreted protein extracts from mutant transmitochondrial cybrids. (**A**) In total, 15 μg of protein extracts from control (CON) and transmitochondrial mutant cybrids (CI^−KD^, CIII^−KO^, CIV1^−KO^, and CIV2^−KO^) were separated on 10% SDS-PAGE gels. GSN levels were analyzed by immunoblotting in whole-cell lysates and isolated mitochondria, using β–actin as the loading control for the whole-cell lysates, and SDHA for the mitochondrial fractions. The signals from four independent experiments per fraction were quantified, normalized by their respective loading controls, and data are presented as the mean ± SD relative to control values, set as 1. GSN, cytosolic GSN; mGSN, mitochondrial GSN. (**B**) In total, 10 μg of protein extracts from whole-cell lysates (WCLs) and mitochondrial fractions (MFs), as well as 35 μg of protein extracts from secreted fractions or secretomes (Ss), were separated by 6% SDS-PAGE. GSN isoforms were analyzed by Western blot. GSN, cytosolic GSN; pGSN, plasma GSN. (**C**) In total, 30 μg of secreted protein extracts were separated on 4–20% TGX^TM^ Precast Protein Gels (Biorad) and pGSN levels were analyzed by immunoblotting. The signals from six independent experiments were quantified, normalized by the total protein amount per lane, and data are presented as the mean ± SD relative to control values, set as 1. Mann–Whitney *U* test: * *p* < 0.05; *** *p* < 0.001.

Then, we analyzed by Western blot and immunoblotting the steady-state levels of cytosolic GSN in whole-cell lysates and in purified mitochondrial fractions from control and mutant cybrids (Figure 2A). GSN signals were quantified and normalized relative to β-actin in whole-cell lysates, and normalized relative to the complex II SDHA subunit in purified mitochondria. Densitometric analyses of four independent experiments confirmed a significant increase of GSN levels in whole cells * (*p* < 0.05), as well as a highly significant increase in the mitochondrial fractions *** (*p* < 0.001) of all mutants relative to control cybrids (Table 3). These results indicate that mGSN levels are preferentially increased in cybrid cells with OXPHOS dysfunction.

### 3.2. Plasma GSN Levels Are Significantly Reduced in the Secretomes from Human Cybrids with OXPHOS Deficiency

Next, we examined the levels of the secreted plasma GSN isoform (pGSN) in extracellular media protein extracts (secretomes) from control and mutant cybrids with OXPHOS deficiency. To confirm this, we identified the specific pGSN isoform in the secretomes from cultured cells, rather than the possible non-specific release of cytosolic GSN in the extracellular media by damaged cells. We first performed a Western blot analysis with an antibody that recognizes both isoforms in whole-cell lysates, mitochondrial fractions, and secretomes from cybrids lacking complex III and an isogenic control (Figure 2B). As expected, the band corresponding to cytosolic GSN (~81 kDa) was predominantly present in the cellular and mitochondrial lysates, and it displayed a slightly lower electrophoretic mobility relative to the pGSN isoform (~86 kDa) observed in the cellular secretomes, compatible with their predicted molecular weights (https://www.uniprot.org/uniprot/P06396). Then, we compared pGSN levels in the secretomes from control and mutant cybrids (Figure 2C) by SDS-PAGE on TGX stain-free^TM^ precast gels (Biorad), followed by Western blot and densitometric analyses of the pGSN signals. The total amount of protein on each lane was used as a loading control for the normalization of pGSN levels, and was calculated by the quantification of fluorescent signals derived from protein tryptophans that react with specific gel trihalo compounds upon UV radiation. Data from six independent experiments revealed a significant decrease of pGSN levels in the secretomes from all mutant cybrids compared to their isogenic controls (Table 3).

### 3.3. Plasma GSN Levels Are Significantly Reduced in Primary Fibroblast Cultures with OXPHOS Deficiency

**Figure 3:**
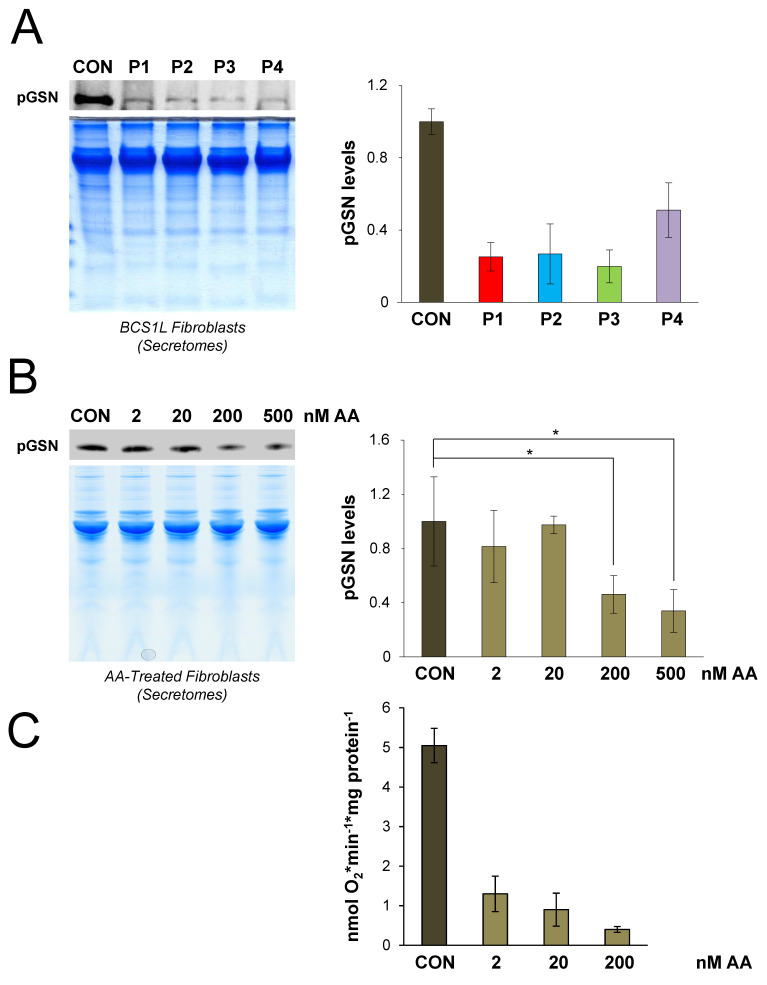
Plasma gelsolin levels are decreased in human fibroblasts with respiratory chain deficiency. (**A**) In total, 25 μg of secreted protein extracts from control (CON) and mutant fibroblasts from patients (P1-P4) harboring pathogenic mutations in the assembly factor BCS1L were separated on 10% SDS-PAGE gels, and pGSN levels were analyzed by immunoblotting. Duplicate gels were stained with Coomassie blue to serve as the loading control (bottom image). The signals from two independent experiments were quantified, normalized by the total protein amount per lane in the Coomassie-stained gels, and data are presented as the mean ± SD relative to control values, set as 1. (**B**) Control fibroblasts were cultured for 48 h in the presence of 2, 20, 200, and 500 nM of antimycin A (AA). In total, 25 μg of secreted protein extracts were separated on 10% SDS-PAGE gels and pGSN levels were subsequently analyzed by Western blot. The signals from four independent experiments were quantified, normalized by the total protein amount per lane, and data are presented as the mean ± SD relative to the control values, set as 1. Mann–Whitney *U* test: * *p* < 0.05. (**C**) Mitochondrial basal oxygen consumption rates (OCR) were analyzed in antimycin A (AA)-treated control fibroblasts respiring in 1 g/L glucose-containing medium. Four technical replicates per sample were measured and data are expressed as the mean ± SD of the untreated cells (CON) values. OCR values were calculated in pmol O_2_.min^−1^.μg protein^−1^ in all experiments.

To exclude that these effects were specific to cybrid cell lines, we tested pGSN levels in the secretomes from primary cultured fibroblasts from patients harboring pathogenic mutations in the complex III assembly factor *BCS1L* (Figure 3A), which previously showed a marked respiratory chain dysfunction [12] together with an upregulated expression and mitochondrial location of GSN (Table 3) [40]. SDS-PAGE gels were stained with Coomassie blue and densitometric measurements per lane were used as loading controls for the normalization of subsequent pGSN immunoreactive signals. Results from two independent experiments revealed a clear trend towards a decrease in pGSN levels in the secretomes from *BCS1L* mutant fibroblasts compared to controls (Figure 3A).

To analyze whether functional rather than structural defects of the OXPHOS system underlie these variations in pGSN levels, we extended our analysis to control fibroblasts treated for 48 h with increasing concentrations (ranging between 2 and 500 nM) of the complex III inhibitor antimycin A (Table 3), which does not affect the assembly or stability of the OXPHOS complexes [40]. Data from four independent experiments showed a significant decrease in pGSN levels only at concentrations of 200 and 500 nM of antimycin A (Figure 3B), which correlated with the decreasing mitochondrial oxygen consumption rates that were below 10% of the mean control value at the highest doses (≥200 nM) of the inhibitor (Figure 3C). Importantly, we previously observed a significant increase of mGSN levels at these two highest inhibitor concentrations [40] (Table 3), again suggesting an inverse correlation between mGSN and pGSN levels in cells with OXPHOS deficiency.

### 3.4. Increased Mitochondrial-to-Plasma GSN Ratio in Human Cellular Models of OXPHOS Deficiency

Altogether, our data indicate that the enrichment of mGSN takes place in parallel to a decreased secretion of the pGSN isoform, and both events seem to arise as a combined response to an enzymatic dysfunction of the OXPHOS system. To establish a numerical correlation that integrates the experimental observations in our different cellular models of OXPHOS deficiency, we calculated the mGSN-to-pGSN protein ratios (Table 3). In mutant cybrids with structural and functional alterations of the MRC complexes, the mGSN:pGSN ratios ranged between ~6- and 11-fold higher than the values measured in their isogenic controls. In fibroblasts with mutations in *BCS1L*, the mGSN:pGSN ratios oscillated between ~4 and 13 times higher than the control values. Additionally, in antimycin A-treated control fibroblasts, the mGSN:pGSN ratios increased gradually in a dose-dependent manner from ~1.3- to 4-fold relative to the untreated cells. Overall, these data indicate that the mGSN-to-pGSN protein ratio is consistently increased in cultured cell lines with a defective respiratory chain, suggesting that it constitutes a bona fide hallmark of OXPHOS dysfunction.

### 3.5. The Mitochondrial-to-Plasma GSN Ratio Can Be Modulated by Genetic Manipulation of OXPHOS-Deficient Cell Lines

**Figure 4:**
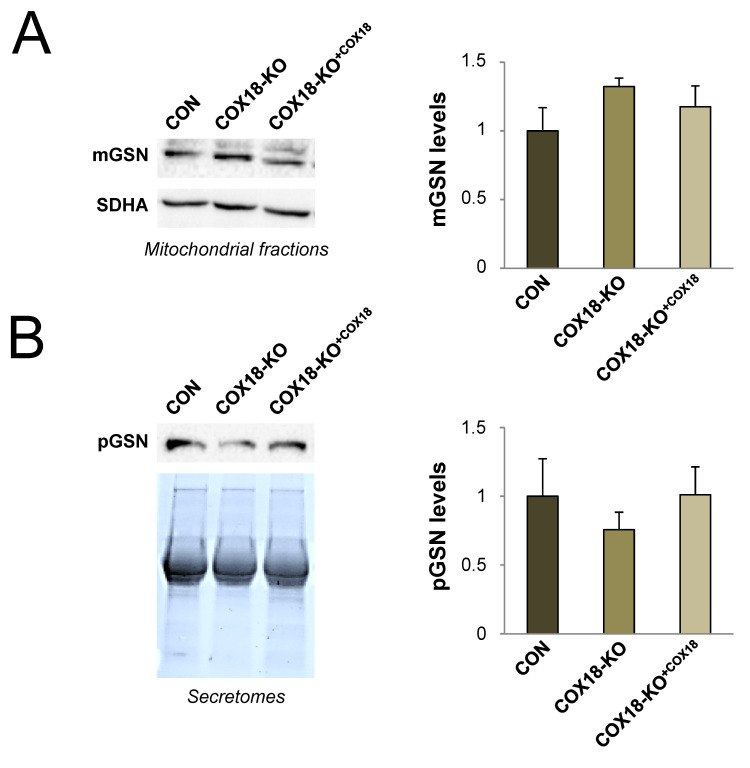
Modulation of the mGSN:pGSN ratio by genetic manipulation of OXPHOS deficiency. Control HEK293T cells (CON, n = 3), and mutant cells depleted of COX18 (COX18-KO, n = 2) and subsequently rescued by stable overexpression of wild-type COX18 (COX18-KO^+COX18^, n = 3) were harvested at 90% confluence. (**A**) In total, 40 μg of mitochondrial protein extracts were separated on 10% SDS-PAGE gels. mGSN levels were analyzed by immunoblotting, using SDHA as the loading control. (**B**) In total, 50 μg of secreted protein extracts were separated on 4–20% TGXTM Precast Protein Gels (Biorad). pGSN levels were analyzed by immunoblotting and normalized by the total protein amount per lane. Experimental data are presented as the mean ± SD relative to control values, set as 1. mGSN, mitochondrial GSN; pGSN, plasma GSN.

Next, we checked whether the mGSN:pGSN ratio was reversibly modulated in response to the genetic manipulation of human HEK293T cells depleted of COX18 (COX18-KO), an assembly factor essential for the biogenesis and function of complex IV [47]. We observed a clear upregulation of mGSN levels in purified mitochondria from COX18-KO cells (Figure 4A), in parallel with decreased pGSN levels in the secretomes (Figure 4B). As a result, the mGSN:pGSN ratio was nearly 2-fold higher in COX18-KO cells than the wild-type control (Table 1). These effects were reversed by stable overexpression of COX18-KO (COX18-KO^+COX18^ cells) (Figure 4C), which recovered the mGSN:pGSN ratio to normal values (Table 3). Therefore, the mGSN:pGSN ratio may change in response to the genetic manipulation of OXPHOS-deficient cell lines.

### 3.6. Oxidative Stress Does Not Trigger the Upregulation and Mitochondrial Location of Cytoplasmic Gelsolin in Human Cybrids

**Figure 5:**
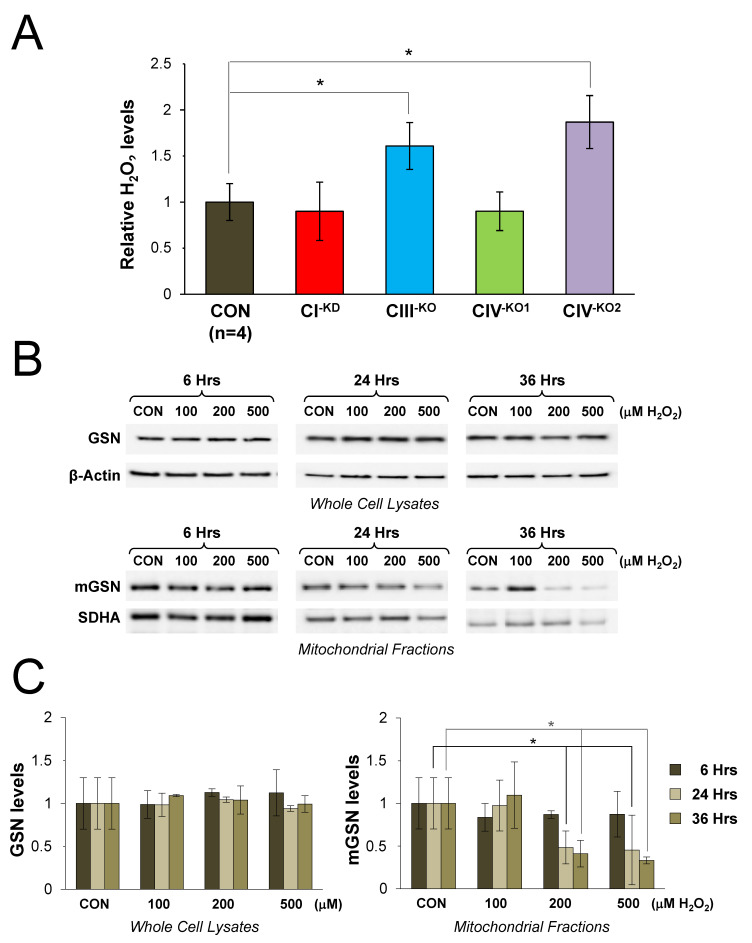
Oxidative stress does not trigger the upregulation and mitochondrial location of gelsolin. (**A**) Hydrogen peroxide levels in isogenic controls (CON, n = 4) and transmitochondrial mutant cybrids (CI^−KD^, CIII^−KO^, CIV1^−KO^, and CIV2^−KO^). Values from eight independent experiments per cell line were normalized by the total protein amount per sample. Data are presented as the mean ± SD of each mutant cell line relative to the mean values of the four isogenic controls, set as 1. Mann–Whitney *U* test: * *p* < 0.05. (**B**) Control cybrids were cultured for 6, 24, and 36 h in the presence of 100, 200, and 500 μM of hydrogen peroxide (H_2_O_2_). In total, 15 μg of whole-cell and mitochondrial protein extracts were separated on 10% SDS-PAGE gels and GSN levels were subsequently analyzed by Western blot, using β–actin as the loading control for the whole-cell lysates, and SDHA for the mitochondrial fractions. (**C**) The signals from four independent experiments were quantified and normalized by their respective loading controls. Data are presented as the mean ± SD relative to the untreated cells (CON) values, set as 1. Mann–Whitney *U* test: * *p* < 0.05. GSN, cytosolic GSN; mGSN, mitochondrial GSN.

We next analyzed whether the observed upregulation and mitochondrial localization of cytosolic GSN was a general phenomenon occurring as a response to any type of oxidative stress, rather than being specific to OXPHOS dysfunction. We first examined the intracellular redox state in control and mutant cybrids with OXPHOS deficiency by measuring endogenous hydrogen peroxide (H_2_O_2_) levels (Figure 5A). Despite the severity of the enzymatic defects in all mutants, the measurements showed significantly increased H_2_O_2_ levels only in the CIII-KO and CIV-KO2 cybrids relative to the controls. In contrast, H_2_O_2_ levels remained within the normal range in the CI-KD and CIV1-KO cybrids, harboring pathogenic homoplasmic mutations in the *MT-ND2* and *MT-CO1* genes, respectively, in agreement with previous observations [53,54]. These results exclude a straightforward correlation between the increased mGSN levels and the varying endogenous H_2_O_2_ levels in the analyzed cell lines.

We next tested whether mGSN expression was upregulated by oxidative stress in a concentration-dependent manner in control 143B-TK^−^ cells treated with increasing concentrations of exogenous H_2_O_2_ (100, 200, and 500 µM) for 6, 24, and 36 h (Figure 5B), as previously shown in HEK293T cells [36]. H_2_O_2_ treatment did not alter GSN steady-state levels in the whole cell lysates but surprisingly led to a significant decrease of mGSN levels in the mitochondrial fractions.

**Figure 6:**
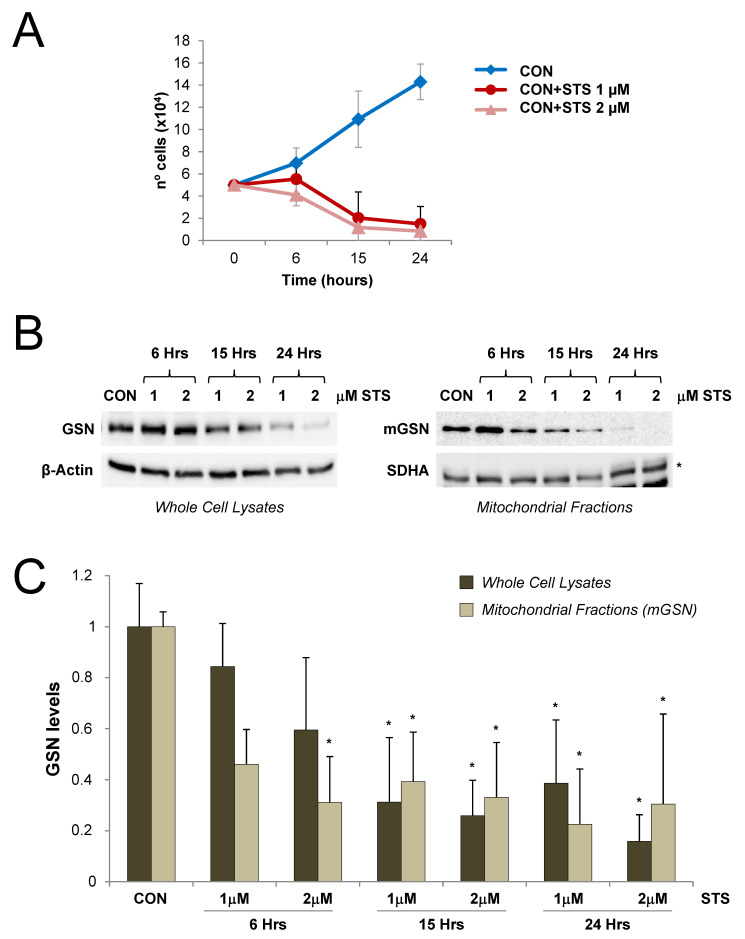
Gelsolin levels decrease upon cell death induction. Control cybrids were cultured for 6, 15, and 24 h in the presence of 1–2 μM of staurosporine (STS). (**A**) Cell death rates upon STS treatment. Here, 5 × 10^4^ cells were seeded in 6-well plates and data are presented as the mean ± SD of three experimental replicates per condition. (**B**) In total, 15 μg of whole-cell and mitochondrial protein extracts were separated on 10% SDS-PAGE gels and GSN levels were subsequently analyzed by Western blot, using β–actin as the loading control for the whole-cell lysates, and SDHA for the mitochondrial fractions (indicated with an asterisk). (**C**) The signals from four independent experiments were quantified and normalized by their respective loading controls. Data are presented as the mean ± SD relative to the untreated cells (CON) values, set as 1. Mann–Whitney *U* test: * *p* < 0.05. GSN, cytosolic GSN; mGSN, mitochondrial GSN.

Similarly, control cells were treated with 1 and 2 μM of the cell death inductor staurosporine (STS) for 6 to 24 h (Figure 6A). Under these conditions, we also observed a significant decrease in mGSN levels (Figure 6B,C) that correlated with cell death (Figure 6A). These data support the notion that H_2_O_2_ treatment may induce protein degradation as a consequence of cell death activation, as previously described [55], and exclude that the upregulation and mitochondrial location of cytosolic GSN primarily responds to alterations in intracellular ROS.

### 3.7. Mitochondrial-to-Plasma GSN Ratio Increases in Blood Samples from Patients with Mitochondrial OXPHOS Disease

Finally, we aimed to determine if a high mGSN:pGSN ratio could be potentially used as a future diagnostic biomarker for OXPHOS dysfunction, by initially comparing blood samples from nine adult patients with mitochondrial OXPHOS disorders (Table 1) relative to nine sex- and age-matched healthy individuals, used as controls (Table 2).

**Figure 7:**
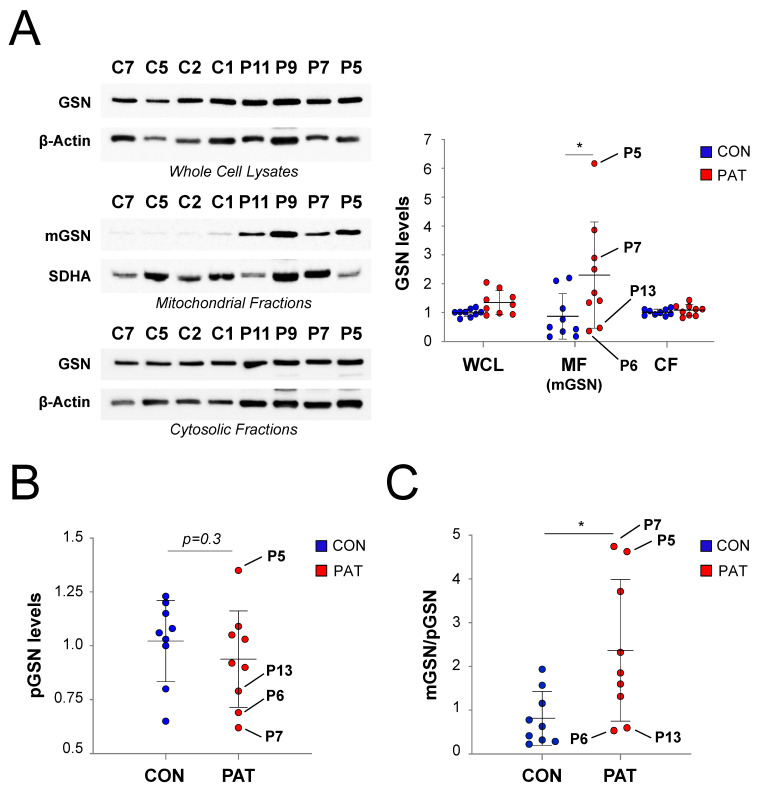
Relative mGSN and pGSN levels in blood samples from patients with mitochondrial OXPHOS disease (MD). (**A**) In total, 15 μg of protein extracts from whole-cell lysate, mitochondrial, and cytosolic fractions from PBMCs isolated from healthy controls (C1–C9, CON) and MD patients (P5-P13, PAT) were separated on 10% SDS-PAGE gels. GSN levels were analyzed by immunoblotting, using β–actin and SDHA as loading controls. (Graph) The signals from three independent biological replicates per fraction were quantified and normalized by their respective loading controls. Each data point represents an individual subject and overall data are presented as the mean ± SD relative to the mean control value. (**B**) Relative pGSN levels from the blood samples of healthy controls (CON) and MD patients (PAT) were determined by ELISA. Each data point represents an individual subject, and overall data are indicated as the mean ± SD from four measurements relative to the mean control value. (**C**) Calculation of the mGSN:pGSN ratios in blood from healthy controls and MD patients. Each data point represents an individual subject, and overall data are indicated as the mean ± SD, relative to the mean control value. The four patients with the most extreme mGSN:pGSN ratios (P5, P6, P7, P13) are indicated. For (**A**–**C**), Mann–Whitney *U* test: * *p* < 0.05. GSN, cytosolic GSN; mGSN, mitochondrial GSN; pGSN, plasma GSN.

To calculate cytosolic GSN levels, we used Western blot followed by GSN immunodetection in whole-cell protein extracts, in cytosolic and in mitochondrial fractions from isolated peripheral blood mononuclear cells (PBMCs), using β-actin and SDHA as loading controls (Figure 7A). Normalized densitometric values revealed significantly increased amounts of mGSN in the mitochondrial fractions, while no significant differences in GSN levels were detected in the remaining PBMC fractions between mitochondrial disease (MD) patients and healthy controls. Interestingly, the mean mGSN value was ~2.5-fold higher in the MD patients than in the healthy individuals (Figure 7A, right graph). To calculate pGSN levels, we used ELISA assays to measure the pGSN levels in plasma samples from the same MD patients. Comparison of the relative pGSN values in MD patients (Table 1) and healthy controls (Table 2) revealed a slight, yet not significant, decrease of pGSN levels in the MD patients when the mean control value was set as 1 (Figure 7B). Based on these results, the average mGSN:pGSN ratio was significantly higher (ranging between ~2.5- and 5-fold) in the MD patients than in controls, with this ratio being well above all control values in four patients (Figure 7C). Unlike our previous observations in the cellular models, mGSN upregulation in PBMCs did not always correlate with a decline of blood pGSN levels in every MD patient (Table 1). These differences probably rise from the wide genetic heterogeneity between the individual healthy donors and patients′ samples, where all individuals are genetically diverse, versus the relative genetic homogeneity of the established cell lines, which allows the establishment of direct comparisons between controls and mutant cells. Therefore, our results, though promising, require further studies to establish the real diagnostic value of the mGSN-to-pGSN protein ratio in blood samples from larger cohorts of healthy controls and patients with mitochondrial OXPHOS disease, as well as of patients with unrelated pathologies.

## 4. Discussion

In this work, we identified a high mGSN-to-pGSN protein ratio as a novel hallmark of OXPHOS dysfunction in human cultured cells. Our data demonstrate that: (1) The cytoplasmic GSN isoform levels are significantly upregulated in the mitochondria from primary fibroblasts and transmitochondrial cybrid cell lines with MRC enzyme deficiency. (2) In parallel, the secreted pGSN isoform levels are significantly decreased in the extracellular environment from human cells with MRC deficiency. (3) The mGSN-to-pGSN ratio values can be modulated by genetic modification of OXPHOS-deficient cell lines. (4) This physiological reverse correlation between both GSN isoforms has not been previously reported in other pathologies. Therefore, we propose to use the ratio between mitochondrial GSN (mGSN) and pGSN levels in the extracellular medium as an indicator of the pathophysiological response to MRC deficiency in cultured cells. This can be extremely helpful, for instance, to differentiate the impact of specific pathogenic mutations or drug treatments in different cultured cell types originally derived from patients, as well as to select for genetically edited cellular models of mitochondrial disorders (MDs). (5) In addition, we provide a proof of principle for future research on the mGSN:pGSN ratio as a potential diagnostics tool for MDs. Since pGSN levels can be easily assessed in blood from patients with unrelated pathologies and healthy individuals [28,56,57,58,59,60], our analyses could be hypothetically extrapolated from cellular models to plasma samples from large cohorts of healthy individuals, as well as of patients with both MDs and unrelated disorders.

Previous 2-D-DIGE proteomics approaches on primary cultured fibroblasts with mutations in the MRC complex III assembly factor BCS1L revealed a significant overexpression of the actin-binding protein GSN in response to complex III deficiency [12]. We next showed that upregulated GSN was primarily located in the mitochondrial fractions from several cellular models with complex III enzymatic or structural defects, where it binds the VDAC pore in the outer mitochondrial membrane to promote antiapoptotic responses [40]. Now, we showed that the increased mGSN levels occur in parallel to a significant decrease in the levels of the secreted pGSN isoform, and that this mechanism is a general response to OXPHOS deficiency with no significant differences among the mitochondrial complexes affected.

In our cellular models, either genetic or enzymatic alterations of the OXPHOS system triggered at least nearly a ~2-fold increase in the mGSN:pGSN ratio values when compared to control cells without MRC impairment, suggesting that mGSN:pGSN ratio values ≥2 would correlate with a poor functioning of the MRC in human cultured cells. However, large differences in the mGSN:pGSN ratio values were observed between mutant fibroblasts, cybrids, and HEK293T cells, which could arise from their different genetic backgrounds and metabolic requirements for ATP synthesis. In highly proliferating cells, like osteosarcoma-derived cybrids or embryonic HEK293T cells, glycolysis accounts for 20–90% of total ATP production, depending on the cell type, with the remainder contributed by mitochondrial oxidation of pyruvate, fatty acids, and glutamine. In contrast, differentiated cells may produce about 95% of the total ATP by OXPHOS and the remaining 5% through aerobic glycolysis [61], which could be the main reason for the largest mGSN:pGSN ratio values in fibroblasts. In addition to their diverse glycolytic capacity, HEK293T cells expressed much lower levels of endogenous GSN than cybrids and fibroblasts, which could also contribute to the differences in the mGSN:pGSN ratio values among these cell types.

Our results suggest that OXPHOS enzymatic dysfunction by itself could be the primary mechanism modulating the expression and localization of both GSN isoforms. These observations, however, do not exclude that the increased mGSN-to-pGSN protein ratios could be a consequence of secondary metabolic or physiopathological alterations induced by MRC enzymatic defects. Among these physiopathological alterations, we considered raised ROS levels to be a likely cause eliciting high mGSN-to-pGSN protein ratios, since oxidative stress was previously reported to increase the expression of cytoplasmic GSN [36]. However, our data do not support this hypothesis for the following reasons: (a) Not all the tested cell lines showed elevated endogenous H_2_O_2_ levels, despite a severe MRC defect associated with a significant upregulation of the mGSN-to-pGSN protein ratios. For instance, the mutant cybrids depleted of MT-CYB [42,62] and MT-CO2 [51] showed increased H_2_O_2_ levels. Similarly, most primary fibroblasts with mutations in *BCS1L* were previously shown to display increased ROS by measuring the amount of oxidized DCFDA to DCF [11]. However, fibroblasts from one patient with the pathogenic p.T50A substitution in BCS1L [11,63] (P4 in our current study) showed normal endogenous H_2_O_2_ levels. In our study, mutant cybrids depleted of MT-ND2 [41] and of MT-CO1 [44] also displayed normal H_2_O_2_ levels, in agreement with previous observations [53]. (b) Control 143B-TK^−^ cells treated with increasing H_2_O_2_ concentrations for up to 36 h actually displayed a significant decrease, rather than an increase, of mGSN levels, more compatible with a general protein degradation as a consequence of cell death activation, as previously described [55]. Similar results were obtained in staurosporine-treated cells, thus excluding a direct correlation between the upregulation of mGSN levels, variations in intracellular ROS levels, and apoptotic cell death. However, we cannot disregard other possible secondary consequences of MRC dysfunction as potential causes of increased mGSN:pGSN protein ratios, including alterations in the mitochondrial membrane potential and ATP synthesis rates [64], or altered oscillations of the intramitochondrial calcium flux [65]. In this regard, it seems especially relevant to gain insight into the interplay between calcium homeostasis and the upregulation of mGSN, as the activity of this protein is regulated by cytosolic calcium [15], and cytosolic GSN expression was shown to be upregulated in human cell lines by treatment with calcium ionophores [35]. In conclusion, our data demonstrate that a high mGSN:pGSN protein ratio is a general response to OXPHOS dysfunction in human cultured cells, but the precise underlying mechanism triggering these cellular adaptations remains open to question.

## Figures and Tables

**Table 1:** Clinical features of 9 adult patients with mitochondrial OXPHOS disease (MD).

Nº	Age	Sex	Genetics	Clinical Phenotype	Enzyme Defect	mGSN	pGSN	mGSN/pGSN
P5	30	M	Single deletion	CPEO	IV	6.23	1.35	4.615
P6	38	F	Single deletion	CPEO + Myopathy	I, III, IV	0.37	0.69	0.536
P7	48	M	*OPA1*	Neuropathy	I	2.93	0.62	4.726
P8	53	M	*POLG*	Exercise intolerance	I (mild)	1.36	1.03	1.320
P9	35	M	*POLG*	Exercise intolerance	III (mild)	2.53	1.09	2.321
P10	58	M	*TK2*	Myopathy	Normal	1.43	0.90	1.589
P11	29	F	*TK2*	Myopathy	I, III, IV	3.91	1.05	3.724
P12	22	M	*MT-CO3* m.9319A>G	Myopathy	I, III	1.70	0.92	1.848
P13	45	M	*MT-TN* m.5688T>C	Myopathy	Normal	0.47	0.79	0.595

Nº: Patient number; M: Male; F: Female; I: Complex I; III: Complex III; IV: Complex IV. Numerical values represent the densitometric signals of mitochondrial GSN (mGSN) quantified in three biological replicates, normalized using SDHA as loading control, and presented relative to the mean mGSN value of control samples (Table 2). Plasma GSN (pGSN) concentrations (μg/mL) were quantified in four experimental replicates and expressed relative to the mean pGSN value of control samples (Table 2).

**Table 2:** Characteristics of 9 healthy adult controls.

Control	Age	Sex	mGSN	pGSN	mGSN/pGSN
C1	22	M	0.306	1.06	0.289
C2	33	M	0.181	0.80	0.226
C3	38	M	1.160	1.00	1.160
C4	53	M	0.505	0.65	0.777
C5	56	M	0.430	1.03	0.417
C6	61	M	0.775	1.23	0.630
C7	33	F	0.349	1.08	0.323
C8	42	F	1.885	1.20	1.571
C9	61	F	2.223	1.15	1.933

M: Male; F: Female. Numerical values represent the densitometric signals of mitochondrial GSN (mGSN) quantified in three biological replicates, normalized using SDHA as loading control, and presented relative to the mean control value. Plasma GSN (pGSN) concentrations (μg/mL) were quantified in four experimental replicates and expressed relative to the mean control value.

**Table 3:** Relative levels of mGSN and pGSN in human cultured cells.

Cell Type	Sample	mGSN	pGSN	mGSN/pGSN
Mutant Cybrids	CON	1 ± 0.40	1 ± 0.20	1
	CI^−KD^	4.66 ± 0.29 ***	0.42 ± 0.23 ***	11.10
	CIII^−KO^	3.70 ± 0.54 ***	0.65 ± 0.09 ***	5.69
	CIV^−KO1^	5.11 ± 0.98 ***	0.76 ± 0.28 *	6.72
	CIV^−KO2^	3.78 ± 1.44 ***	0.56 ± 0.29 ***	6.75
	CON	1 ± 0.25	1 ± 0.07	1
	P1	2.40 ± 1.00	0.25 ± 0.08	9.60
*BCSL1* Mutant	P2	2.27 ± 0.31	0.27 ± 0.17	8.41
Fibroblasts ^¶^	P3	2.60 ± 0.94	0.20 ± 0.09	13.00
	P4	2.23 ± 0.64	0.51 ± 0.15	4.37
	CON	1 ± 0.25	1 ± 0.33	1
	2 nM AA	1.07 ± 0.25	0.81 ± 0.27	1.32
AA-treated	20 nM AA	1.16 ± 0.33	0.76 ± 0.38	1.53
Fibroblasts ^¶^	200 nM AA	1.35 ± 0.35 *	0.67 ± 0.26 *	2.02
	500 nM AA	1.39 ± 0.34 ***	0.34 ± 0.16 *	4.09
HEK 293T Cells	CON	1 ± 0.17	1 ± 0.27	1
	COX18^−KO^	1.32 ± 0.06	0.76 ± 0.13	1.75
	COX18^−KO+COX18^	1.18 ± 0.15	1.01 ± 0.20	1.16

Relationship between mGSN and pGSN levels quantified in transmitochondrial mutant cybrids, complex III-deficient fibroblasts with mutations in *BCS1L*, antimycin A (AA)-treated control fibroblasts, and genetically modified HEK293T cells. ^¶^ The relative mGSN levels were calculated in reference [39]. Numerical values represent the mean ± SD to the mean control (CON) value (set as 1) of four independent experiments. Mann–Whitney *U* test: * *p* < 0.05; *** *p* < 0.001.
